# Pneumatosis cystoides intestinalis: six case reports and a review of the literature

**DOI:** 10.1186/s12876-018-0794-y

**Published:** 2018-06-28

**Authors:** Yong juan Wang, Yu ming Wang, Yan min Zheng, Hui qing Jiang, Jie Zhang

**Affiliations:** 1 Department of Gastroenterology and Hepatology, The Second Affiliated Hospital of Hebei Medical University, Hebei, China; 2 Department of Gastroenterology and Hepatology, The General Hospital of Tianjin Medical University, Tianjin, China

**Keywords:** Pneumatosis Cystoides intestinalis, Diagnosis, Endoscopy, Therapy

## Abstract

**Background:**

Pneumatosis cystoides intestinalis (PCI) is characterized by gas-filled cysts in the intestinal submucosa and subserosa. There are few reports of PCI occurring in duodenum and rectum. Here we demonstrated four different endoscopic manifestations of PCI and three cases with intestinal stricture all were successfully managed by medical conservative treatment.

**Case presentation:**

There are 6 cases of PCI with varied causes encountered, in which the etiology, endoscopic features, treatment methods and prognosis of patients were studied. One case was idiopathic, while the other one case was caused by exposing to trichloroethylene (TCE), and the remaining four cases were secondary to diabetes, emphysema, therioma and diseases of immune system. Of the six patients, all complained of abdominal distention or diarrhea, three (50%) reported muco-bloody stools, two (33.3%) complained of abdominal pain. In four other patients, PCI occurred in the colon, especially the sigmoid colon, while in the other two patients, it occurred in duodenum and rectum. Endoscopic findings were divided into bubble-like pattern, grape or beaded circular forms, linear or cobblestone gas formation and irregular forms. After combination of medicine and endoscopic treatment, the symptoms of five patients were relieved, while one patient died of malignant tumors.

**Conclusion:**

PCI endoscopic manifestations were varied, and radiology combined with endoscopy can avoid misdiagnosis. The primary bubble-like pattern can be cured by endoscopic resection, while removal of etiology combined with drug therapy can resolve majority of secondary cases, thereby avoiding the adverse risks of surgery.

## Background

Pneumatosis cystoides intestinalis (PCI) is characterized by gas-filled cysts in the intestinal submucosa and subserosa. It is a rare disease with reported incidence of 0.03% and can occur in any age group [1]. PCI was first described by DuVernoi in 1783 and later subcategorized by Koss in 1952 [2]. It can be divided into primary or idiopathic (15%) type, which refers to air pockets that are cystic in appearance and imply to a chronic, benign idiopathic etiology [3], and secondary type (85%), which refers to radiological findings of linear, microvesicular, or more circumferential appearing intramural gas caused by various predisposing factors [3, 4]. The pathogenesis of PCI remains unclear; however, six pathophysiologic mechanisms have been proposed including inflammation, physical damage of intestinal mucosa, nutritional imbalance and dysbacteriosis, gastrointestinal dysmotility, and immune dysfunction [5, 6]. PCI can occur anywhere within the gastrointestinal tract from the esophagus to the rectum. It was previously reported that mainly involve the terminal ileum, but Morris later reported that PCI localized to large intestine in 46% of the cases, the small intestine in 27% of the cases, the stomach in 5% of the cases and the large combined with small intestine in 7% of the cases [7]. PCI can be found incidentally in asymptomatic patients, while some cases presented as abdominal pain, diarrhea, abdominal distention, constipation, bloody stool, flatus, loss of appetite, weight loss, and even life-threatening illnesses including bowel necrosis and perforation [8].

Several reports of endoscopic ultrasonography (EUS) in the evaluation of bumps in the colon have clarified the diagnosis of PCI [9, 10], while computed tomography scan is regarded as the most sensitive imaging modality for detection. However, some patients who frequently presented with non-specific gastrointestinal symptoms were often prone to be misdiagnosed or maltreated and even underwent surgical resection, which resulted in several adverse events. Herein, we reported the etiological characteristics, endoscopic features, treatment and prognosis of 6 PCI cases to recognize the endoscopic characteristics and investigate the proper treatment methods for PCI.

## Case presentation

### Case 1

**Fig. 1:**
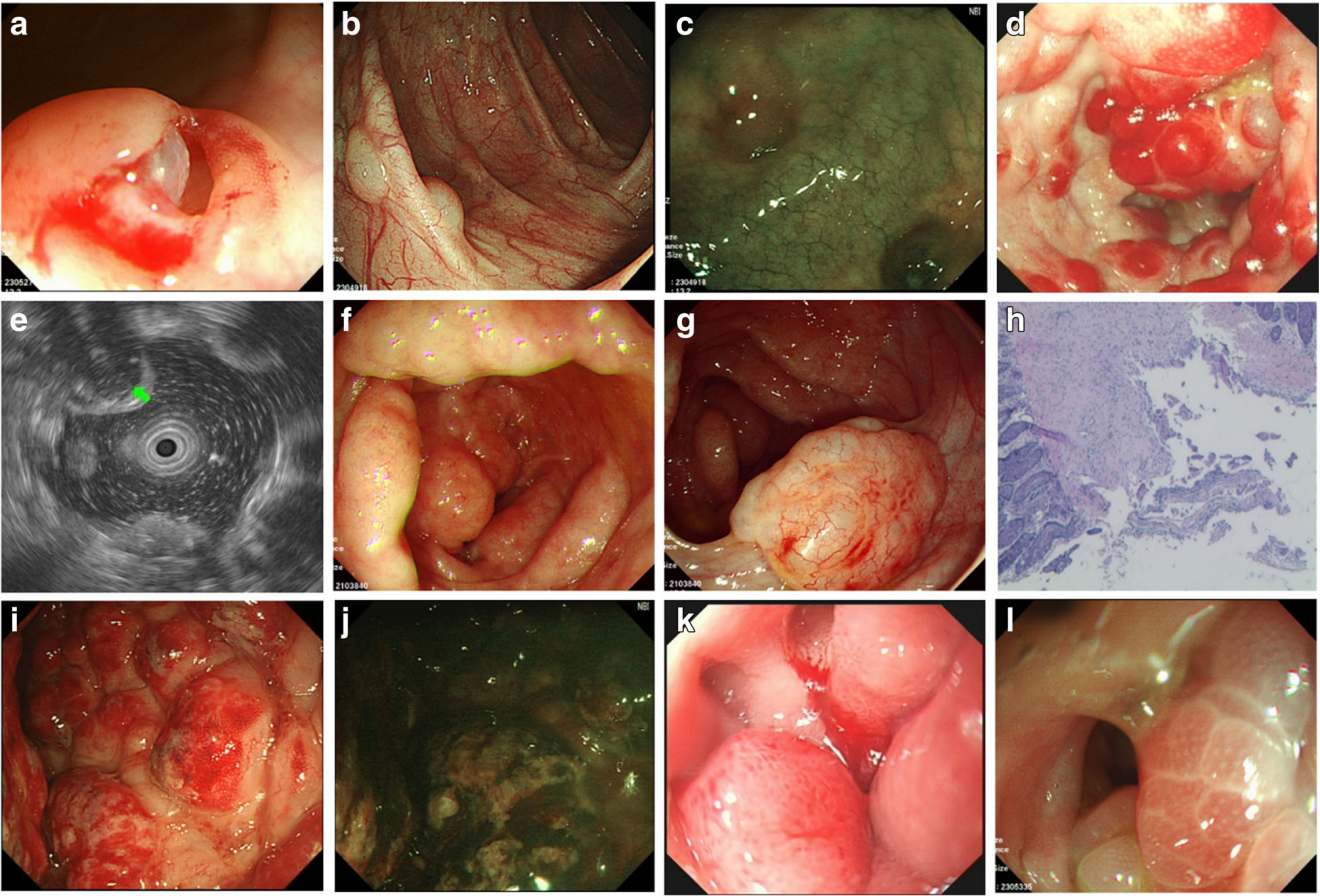
Endoscopic features of PCI. Case 1: (**a**-**c**); Case 2: (**d**, **e**); Case 3: (**f**-**h**); Case 4: (**i**, **j**); Case 5: (**k**); Case 6: (**l**). **a** Biopsies were done to reveal the nature of the cystic lesions underneath the mucosa; **b** Scattered bubble-like or cystoid nodules were seen, which needed to be distinguished from polyps; **c** NBI showed clear texture of intestinal wall vessels; **d** Grape-like or beaded subepithelial lesions were identified in the colon, some with erythematous mucosa; **e** The endoscopic ultrasonography showed low echo of cystic below the mucosal layer; **f** Line or pebble like sessile cysts were distributed around the colon, with normal overlying mucosa; **g** Irregular forms were disclosed, which needed to be distinguished from malignant tumor; **h** The pathologic findings revealed submucosal cystic structure; **i** Irregular forms needed to be distinguished from Crohn’s disease; **j** NBI showed unclear vascular texture on mucosal surface; **k** Gastroscope demonstrated duodenal gas cyst, leading to lumen stenosis; **l** Appearance of air cysts in the rectum, honeycomb-like

A 56-year-old man was admitted to the hospital with abdominal distension and diarrhea for several days. Physical examination revealed no abnormality. Routine laboratory examinations, bacterial and parasitic stool examinations and viral serology were negative. Chest and abdomen X-ray showed no obvious abnormality. However, endoscopic examination disclosed scattered bubble-like or cystoid nodules, which distributed in transverse colon (Fig. 1b). Meantime, narrow band imaging (NBI) showed clear texture of intestinal wall vessels (Fig. 1c). Considered PCI was idiopathic, we used high frequency electrosurgical resection of the gas cysts, and the cysts were collapsed after the gas was discharged (Fig. 1a). The patient was treated with Bifidobacterium (420 mg/bid) to improve his intestinal function (Table 1). We also advised him to eat less gas-producing foods without using any antibiotic. The patient was symptom-free after one week and the lesions disappeared completely after three months of follow-up (Fig. 3a, b).

**Table 1:** General conditions, clinical manifestations, endoscopic features, treatment and prognosis of patients with PCI

No.	Age	Gender	symptoms	Etiology	Loc.	Endoscopic findings	PCI treatment	Outcome	Follow-up (mo)
1	56	M	abdominal distension diarrhea	–	Transverse ascending	bubble-like	Bifidobacterium endoscopic treatment	remission	3
2	48	F	Constipation, diarrhea, muco-bloody stools, abdominal distension farting more	exposure to TCE (1 year)	Sigmoid descending	grape or beaded	tinidazole, Bifidobacterium endoscopic treatment	remission	4
3	66	F	constipation, diarrhea muco-bloody stools abdominal pain	AA (20 years) UCTD (2 years)	Sigmoid	linear or bullous	Bifidobacterium aluminum phosphate	remission	10
4	72	F	constipation, diarrhea muco-bloody stools abdominal distension farting more	diabetes (10 years)	Sigmoid ascending	irregular forms	rifaximin metronidazole Bifidobacterium	remission	6
5	64	M	abdominal distension abdominal pain	emphysema (1 year)	Duodenal- bulb	bubble-like	Bifidobacterium	remission	8
6	27	F	abdominal distension diarrhea abdominal pain	peritoneal carcinoma	Rectum	grape or beaded	Bifidobacterium	died	

*TCE* trichloroethylene, *AA* aplastic anemia, *UCTD* Undifferentiated connective tissue disease

### Case 2

**Fig. 2:**
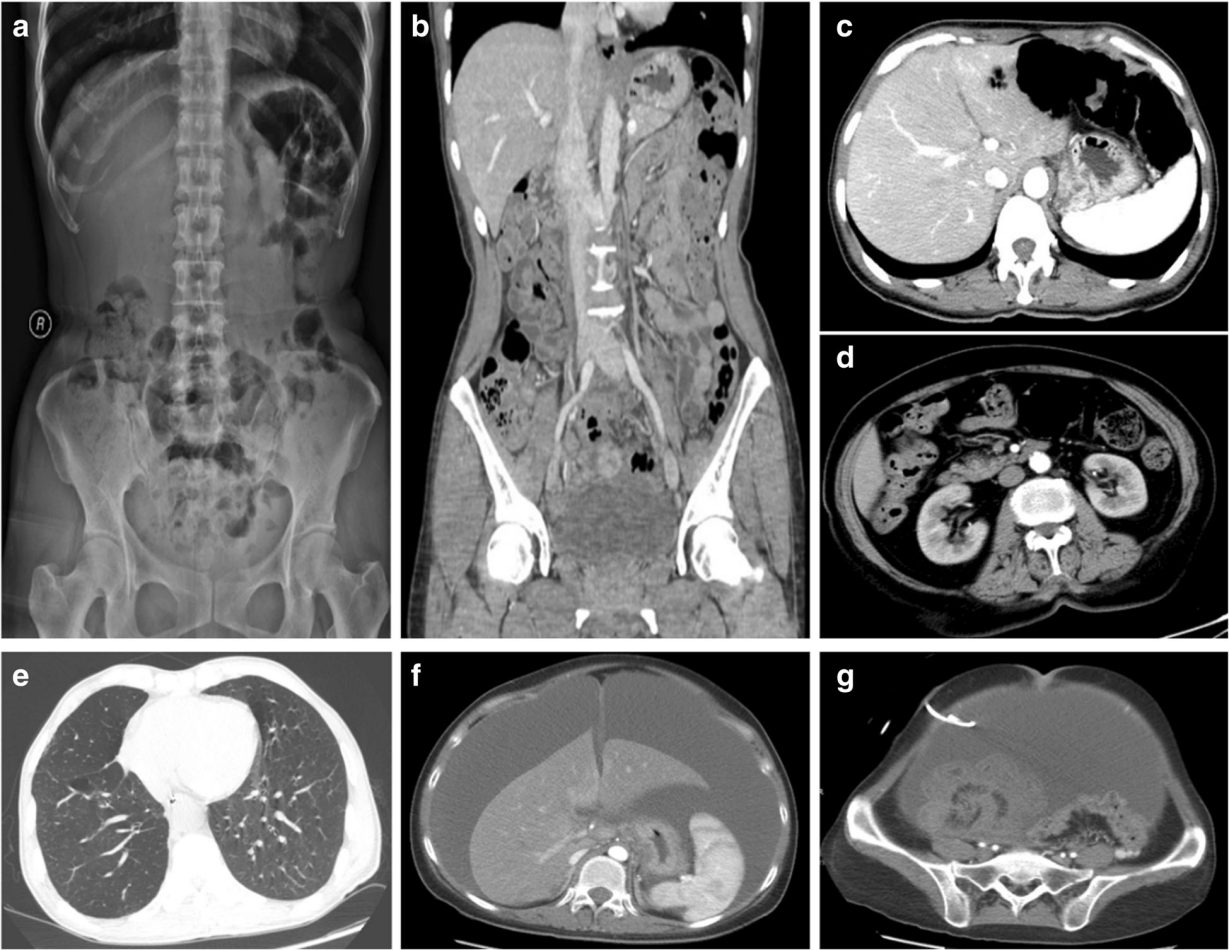
Imaging findings. Case 2: (**a**, **b**); Case 3: (**c**); Case 4: (**d**); Case 5: (**e**); Case 6: (**f**, **g**). **a** Abdominal X-ray showed multiple intraluminal gas pockets in the left colon; **b** Coronal reconstruction confirmed multiple submucosal lesions; **c** Abdominal CT showed no portal venous gas embolism. **d** Abdominal CT revealed multiple polypoid lesions of the colon; **e** Chest CT showed centrilobular emphysema, pulmonary field scattered in small circular distribution. **f** Abdominal CT showed a large presence of ascites in the abdomen. **g** Pelvic CT demonstrated primary peritoneal carcinomatosis with balloon like structures in the rectum

**Fig. 3:**
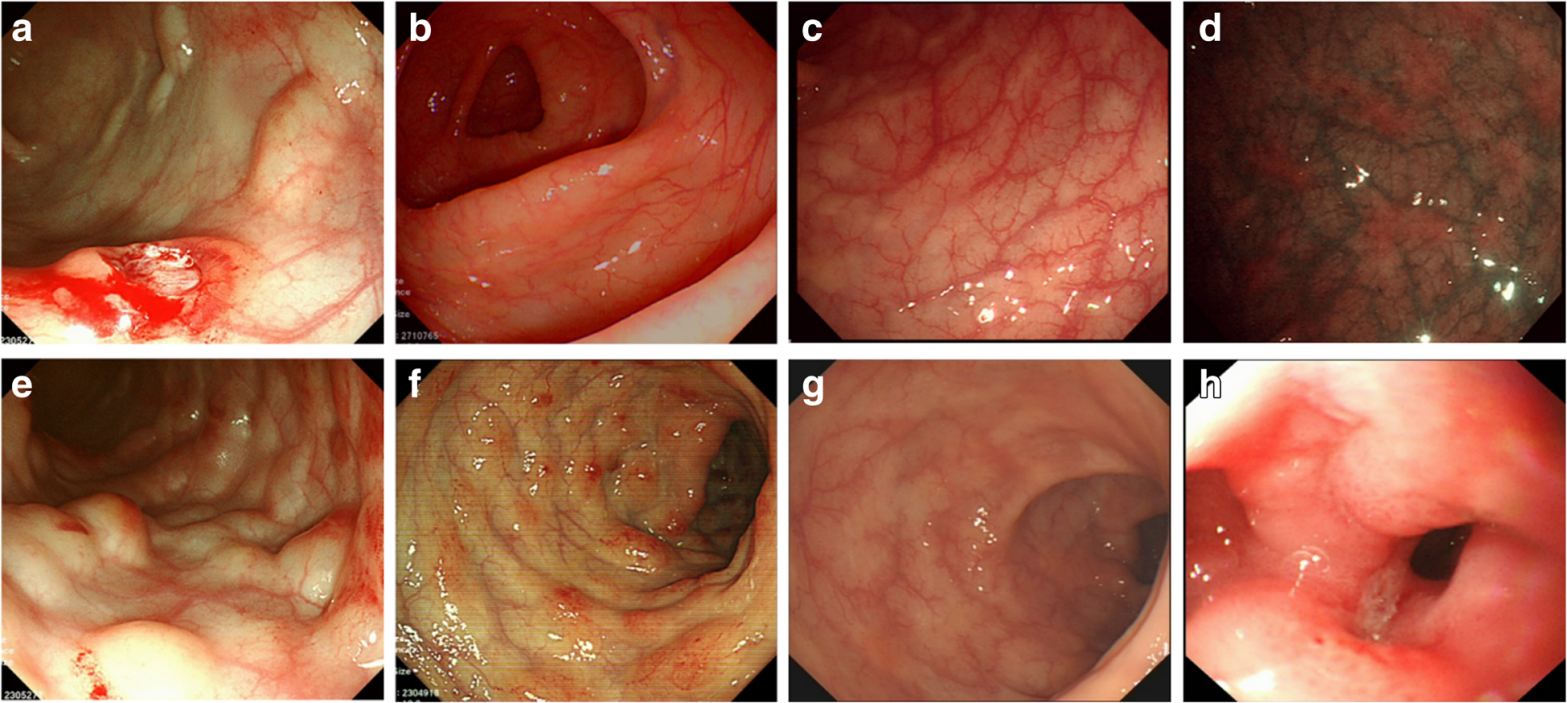
Endoscopic manifestations after treatment. Case 1: (**a**, **b**); Case 2: (**c**, **d**); Case 3: (**e**); Case 4: (**f**, **g**); Case 5: (**h**). **a** Biopsies were performed, with the release of “air” and cyst collapse; **b** Gas cyst completely disappeared after treatment; **c** The cyst of the colon disappeared; **d** NBI demonstrated visible patchy erythema and yellow nodules; **e** Gas-filled cysts flattened; **f** The cyst of the colon improved, but mucosal surface redness still existed; **g** Colonic gas cyst was full recovery; **h** Duodenal descending presented as stenosis due to gas cyst

A 48-year-old woman complained of constipation for 5 days, and then turned into diarrhea with discontinuous abdominal distension. She was hospitalized because of severe diarrhea (7 times/ d) with muco-bloody stools for one week. The stool frequency was five times a day. Her previous medical history revealed exposure to trichloroethylene (TCE) for one year. (Table 1) Physical examination at admission revealed extensive abdominal tenderness. Fecal occult blood tests were positive. Other serological markers for autoimmunity and viral serology were normal, as was stool examination for bacteria and parasites. However, abdominal X-ray showed multiple intraluminal gas pockets in the left colon (Fig. 2a). Coronal reconstruction confirmed the widespread serosal intestinal air cysts involving long segment of colon (Fig. 2b). Colonoscopy revealed grape-like or beaded subepithelial lesions, and some with erythematous mucosa distributed in the sigmoid. Given the narrowing of the lumen secondary to these lesions the colonoscopy was incomplete (Fig. 1d). The endoscopic ultrasonography showed low echo of cystic below the mucosal layer (Fig. 1e). We used high frequency electrosurgical resection of the gas cysts; But considering that extensive endoscopic therapy might lead to infection, we used only partial treatment. Since the narrow lumen of the patient, we restricted her food intake and used parenteral nutrition. One week later, the patient started to have a half-fluid diet. Ornidazole (500 mg/bid) and vitamin B2 (10 mg/bid) were given to regulate intestinal anaerobes, while bifidobacterium (420 mg/bid) was given at intervals of half an hour. We also advised him to eat less gas-producing foods. The patients’ condition improved after 2 weeks (Table 1). One month later the lesions disappeared completely (Fig. 3c) and NBI demonstrated visible patchy erythema and yellow nodules (Fig. 3d). After four months of follow up, the patient was still no symptoms, and findings at colonoscopy were normal.

### Case 3

A 66-year-old woman was admitted to the gastroenterology ward because of alternate constipation and diarrhea with muco-bloody stools. She had a history of undifferentiated connective tissue disease (UCTD) for 20 years and aplastic anemia (AA) for 1 year. In the past, she was mainly treated with glucocorticoid, and subsequently developed AA. Recently she presented with a diffuse pain in the abdomen with muco-bloody stools. Physical examination at admission revealed extensive abdominal tenderness. The biochemical tests revealed cytopenia due to AA and no obvious abnormality in stool culture for pathogens. Blood cultures were also negative. Computer tomography (CT) examination showed no portal venous gas embolism (Fig. 2c). Colonoscopic examination disclosed line or pebble like sessile cysts and irregular forms, which mainly distributed in sigmoid (Fig. 1f, g). Irregular forms of PCI with large bulge should be distinguished from malignant tumor (Fig. 1g). Given the narrowing of the lumen secondary to these lesions, the colonoscopy was incomplete. The pathologic findings revealed submucosal cystic structure (Fig. 1h). We used high frequency electrosurgical resection of the gas cysts. Because of the history of AA, she was treated with aluminum phosphate (20 g/bid) and bifidobacterium (420 mg/bid) without any antibiotics. We also advised him to eat less gas-producing foods. Symptoms of diarrhea improved significantly after one month, and gas-filled cysts became flattened (Fig. 3e). After ten months of follow-up the clinical symptoms were still resolved.

### Case 4

A 72-year-old woman complained of constipation for several days, and then turned to diarrhea for one month. She was hospitalized for muco-bloody stools and severe abdominal distension. She had a history of diabetes for 10 years and was mainly treated with acarbose and insulin. Physical examination at admission revealed extensive abdominal tenderness. Routine laboratory examinations, bacterial and parasitic stool examinations and viral serology were negative. Abdominal X-ray showed multiple intraluminal gas pockets in sigmoid and ascending colon. Computer tomography (CT) examination showed multiple polypoid lesions of the colon (Fig. 2d). Colonoscopy showed irregular forms of lesions that were covered with mucosa of normal appearance in sigmoid and ascending colon (Fig. 1i). Irregular forms of PCI and mucosal lesions with erosion should be distinguished from Crohn’s disease. Given the narrowing of the lumen secondary to these lesions, the colonoscopy was incomplete. NBI showed mucosal redness, which appeared as punctate labelling, and the blood vessels of intestine were clear (Fig. 1j). We used high frequency electrosurgical resection of the gas cysts. She was banned from using acarbose, continued to use insulin treatment for diabetes. The patient was initially treated with ornidazole (500 mg/bid) and bifidobacterium (420 mg/bid). In view of the older age of the patient and a history of diabetes, the antibiotic was changed to rifaximin (200 mg/qid) to avoid antibiotic resistance. We also advised him to eat less gas-producing foods. The patients’ condition was improved after one month and the findings at endoscopy were improved (Fig. 3f). After 6 months of follow up, the cysts gas disappeared (Fig. 3g).

### Case 5

A 64-year-old man was admitted to the hospital with wheezes and exertional dyspnea, which he had suffered from for several months, but without abdominal symptoms. He was diagnosed with emphysema pulmonum 1 years ago. Physical examination revealed double diffuse rales. Serological markers for autoimmunity and viral serology were normal, so as was the stool examination. Chest CT showed centrilobular emphysema, pulmonary field scattered in small circular distribution (Fig. 2e). A chest X-ray and CT examination showed the improvement of emphysema pulmonum. Endoscope showed stenosis due to gas cyst in duodenal descending (Fig. 1k). Emphysema as the primary disease, it is mainly used seretide for treatment. The PCI was treated successfully with intensive (but not hyperbaric) oxygen therapy and bifidobacterium (420 mg/bid). The findings at endoscopy were improved after eight months of follow-up (Fig. 3h).

### Case 6

A 27-year-old woman was hospitalized because of ascites and abdominal pain for 3 months. She was diagnosed as Budd-Chiari syndrome (BCS) before admission. Physical examination at admission revealed pronounced abdominal tenderness and abdominal mass. Routine laboratory examinations, bacterial and parasitic stool examinations and viral serology were negative, while the level of serum C125 increased significantly up to 1560 U/ml. Abdominal X-ray and Abdominal CT showed a large presence of ascites in the abdomen (Fig. 2f). CT showed massive hydrops of abdominal cavity, multiple intraluminal gas pockets in the rectum and ovarian mass (Fig. 2g), which was limited to the ovarian surface without invasion. Abdominal ultrasonography revealed massive hydrops in the abdominal cavity, and abdominal paracentesis indicated bloody ascites. Colonoscopy showed grape or beaded lesions (Fig. 1l). Finally, primary peritoneal carcinoma (PPC) was diagnosed by peritoneal biopsy. After this the patient was given nutritional support. Finally, she was transferred to a hospital near her home and unfortunately died.

## Discussion and conclusion

PCI is a rare condition characterized by gas-filled cysts in the intestinal submucosa and subserosa. Various predisposing factors have been associated with PCI: postsurgery [11], chemotherapy [12], Acarbose [13], Trichloroethylene (TCE) [14], scleroderma [15], and pulmonary illness [16]. In this report, there were total 6 patients studied, which included one case of idiopathic, one case induced by TCE, one case with UCTD, one case induced by emphysema, one case caused by diabetes and one case with PPC. Exposure to TCE results in primary PCI that previously reported in Japan, and the pathogenesis may be related to intestinal mucosal toxicity [14]. Patients with UCTD can be combined with PCI, including systemic sclerosis (SS), systemic lupus erythematosus (SLE), etc., which may be related to abnormal immune function regulation [17] and long-term use of glucocorticoids resulted in intestinal mucosal atrophy and defects, which promoted the formation of cysts in the intestinal submucosa. PCI has been associated with pulmonary disease in the absence of gastrointestinal disorder [16]. Recently, several reports have mentioned that the α-GI, voglibose or Acarbose, might be a causative factor for PCI [13, 18, 19]. It is generally understood that α-GI causes flatulence, because it suppresses absorption of carbohydrates in the colon, and intestinal bacteria then generates a large volume of gas by carbohydrate fermentation [20]. Recently, application of rituximab in patients with squamous cell carcinoma [21], sunitinib in patients with renal cell carcinoma, gefitinib in patients with lung adenocarcinoma and chemotherapy drugs, such as fluorouracil in patients with colorectal cancer were suggested to be associated with the development of PCI [22]. In this study, one of patient was suffering from peritoneal cancer, but did not use chemotherapy drugs, so the relationship between the tumor and the intestinal gas cyst requires further study. Recently, molecular-targeted agents such as anti-vascular endothelial growth factor (VEGF) agents and epidermal growth factor receptor (EGFR) tyrosine kinase inhibitors have been suspected to increase intestinal toxicity as well, which is interrelated to PCI [23].

Patients with PCI can be asymptomatic or present with non-specific gastrointestinal symptoms such as abdominal distention, diarrhea, constipation, abdominal pain and weight loss [5]. Although PCI was previously reported to occur more frequently in the terminal ileum of the gastrointestinal tract, Horiuchi A’s studies showed that PCI occurred 61.8% in colon,15.4% in the small intestine, which was more common in the sigmoid colon [24]. In this study, in 4 (66.7%) patients, PCI occurred in the colon, especially the sigmoid colon, while in other two (33.3%) patients, it occurred in duodenum and rectum, which was relatively rare. We looked up a lot of literature and found that there were fewer reports of PCI occurred in duodenum and rectum, so more clinical evidence needed further study. Since the lack of specificity in clinical manifestations, it should be differentiated from the duodenal diverticulum when PCI occurred in that duodenum. It has been reported that PCI can be found in mesentery, omentum, ligamentum hepatogastricum, but rarely found in esophagus and stomach. Endoscopic findings were divided into bubble-like pattern, grape or beaded circular forms, linear or cobblestone gas formation and irregular forms. The bubble-like lesion was often idiopathic, which needed to be distinguished from polyps. The grape-like or beaded subepithelial lesions should be differentiated from intestinal tuberculosis. Irregular shapes need attention to distinguish from tumors and Crohn’s disease. In previously described cases, linear gas formations were an ominous sign [25]. The radiological findings of diffuse and extensive intraluminal cysts within the walls of the colon are classic for PCI. Contrast-enhanced CT has an advantage in revealing gas within the portal venous system [26]. Endoscopic ultrasonography findings of the mucosal layer low echo below the cystic in the diagnosis of PCI is superior [27], which can avoid the risk of CT examination and distinguish from other diseases, such as polyps, carcinoma, lymphoma, liquid cysts, Gardner syndrome, Cronkhite-Canada syndrome, Peutz-Jehers syndrome, Crohn’s disease, etc. [28]. Overall, the diagnosis of PCI and its adverse events including bowel perforation, portal venous gas (PVG) [29], intestinal hemorrhage, sepsis or peritonitis is very important.

PCI is a benign lesion and very few patients face cancer risk. There is no unified standard for treatment of PCI. The most of PCI cases are usually managed conservatively, while exploratory laparotomy is considered if peritoneal irritation or persistent bowel obstruction occurs in PCI. Joseph D. Feuerstein proposed that PCI could be effectively alleviated with hyperbaric oxygen therapy at 2.5 atmospheres for 2.5 h for at least three sessions [30, 31]. In our cases, we gave oxygen therapy to PCI patients with emphysema, but without hyperbaric oxygen, which can alleviate the patient’s COPD, meanwhile regarded as the treatment methods of PCI associated with emphysema. Electronic colonoscopy forceps can facilitate the treatment of bubble-like pattern PCI, however blindly using argon knife excision may cause an explosion considering the possible existence of methane and other combustible gases. In addition, ornidazole and Bifidobacterium can be used to regulate intestinal flora, which can obviously improve the symptoms. The mechanism of antibiotic therapy is to inhibit the growth of harmful intestinal bacteria and thus to inhibit the production of hydrogen. The application of Bifidobacterium is to regulate the dysbacteriosis and improve the function of gastrointestinal tract. Knechtle reported that low pH (< 7.3), low serum bicarbonate (< 20 mmol/L) and elevated serum lactic acid (LA) (> 2 mmol/L) are associated with poor prognosis [32]. Alexander J found that surgical treatment of PCI patients with PVG could decrease the risk of death as compared to other PCI patients, so he recommended that surgical treatment was needed if the patient was not responsive to conservative treatment or had developed serious adverse events. Through the study of 123 cases of PCI, Ho-Su Lee et al. concluded that patients with both peritoneal irritation and decreased or absent enhancement of bowel wall on CT should be observed, and we should establish a simple and novel risk score that predicted mortality in patients with PCI [33]. The treatment of primary diseases is imperative for secondary PCI. Long-term follow-up of patients with PCI will facilitate understanding of the disease prognosis.

In this article, we demonstrated 6 cases of PCI with varied causes and 3 of 6 cases with intestinal stricture all were successfully managed by medical conservative treatment. PCI endoscopic manifestations were varied, and radiology combined with endoscopy could avoid misdiagnosis. The primary bubble-like pattern can be cured with endoscopic resection, while removal of etiology combined with drug therapy can resolve majority of secondary cases, thereby avoiding the adverse risks of surgery.

## Abbreviations

AA: Aplastic anemia
BCS: Budd-Chiari syndrome
CT: Computer tomography
EGFR: Epidermal growth factor receptor
EUS: Endoscopic ultrasonography
LA: Lactic acid
PCI: Pneumatosis cystoides intestinalis
PPC: Primary peritoneal carcinoma
PVG: Portal venous gas
SLE: Systemic Lupus erythematosus
SS: Systemic sclerosis
TCE: Trichloroethylene
UCTD: Undifferentiated connective tissue disease
VEGF: Anti-vascular endothelial growth factor
